# Role of the Stress Index in Predicting Mortality among Patients with Traumatic Femoral Fractures

**DOI:** 10.3390/diagnostics14141508

**Published:** 2024-07-12

**Authors:** Ching-Ya Huang, Sheng-En Chou, Chun-Ying Huang, Ching-Hua Tsai, Shiun-Yuan Hsu, Ching-Hua Hsieh

**Affiliations:** 1 Department of Plastic Surgery, Kaohsiung Chang Gung Memorial Hospital, College of Medicine, Chang Gung University, Kaohsiung 83301, Taiwan; iyaaa1114@gmail.com; 2 Department of General Surgery, Kaohsiung Chang Gung Memorial Hospital, College of Medicine, Chang Gung University, Kaohsiung 83301, Taiwan; athenechou@gmail.com; 3 Department of Trauma Surgery, Kaohsiung Chang Gung Memorial Hospital, College of Medicine, Chang Gung University, Kaohsiung 83301, Taiwan; junyinhaung@cgmh.org.tw (C.-Y.H.); tsai1737@cloud.cgmh.org.tw (C.-H.T.); ah.lucy@hotmail.com (S.-Y.H.)

**Keywords:** traumatic femoral fracture, stress index (SI), glucose, potassium, mortality

## Abstract

Background: Traumatic femoral fractures, often resulting from high-energy impacts such as traffic accidents, necessitate immediate management to avoid severe complications. The Stress Index (SI), defined as the glucose-to-potassium ratio, serves as a predictor of mortality and adverse outcomes in various trauma contexts. This study aims to evaluate the prognostic value of the SI in patients with traumatic femoral fractures. Methods: This retrospective cohort study included adult trauma patients aged 20 or above with traumatic femoral fractures from the Trauma Registry System at a level 1 trauma center in southern Taiwan between 1 January 2009 and 31 December 2022. At the emergency room, serum electrolyte levels were assessed using baseline laboratory testing. By dividing blood glucose (mg/dL) by potassium (mEq/L), the SI was calculated. The best cut-off value of the SI for predicting mortality was determined using the Area Under the Curve (AUC) of Receiver Operating Characteristic (ROC). Results: A total of 3717 patients made up the final group, of which 3653 survived and 64 died. In comparison to survivors, deceased patients had substantially higher blood glucose levels (199.3 vs. 159.0 mg/dL, *p* < 0.001) and SIs (53.1 vs. 41.6, *p* < 0.001). The optimal SI cut-off value for predicting mortality was 49.7, with a sensitivity of 53.1% and a specificity of 78.7% (AUC = 0.609). High SI was associated with increased mortality (4.2% vs. 1.0%, *p* < 0.001) and longer hospital stays (12.8 vs. 9.5 days, *p* < 0.001). The adjusted odds ratios of mortality, controlled by comorbidities, the Glasgow Coma Scale, and the Injury Severity Score, were significantly higher in patients with a higher SI (AOR 2.05, *p* = 0.016) than those with a lower SI. Conclusions: Elevated SI upon admission correlates with higher mortality and extended hospital stay in patients with traumatic femoral fractures. Although the SI has a moderate predictive value, it remains a useful early risk assessment tool, necessitating further prospective, multi-center studies for validation and standardization.

## 1. Introduction

Traumatic femoral fractures, often resulting from high-energy impacts such as traffic accidents, predominantly affect young males and require immediate, precise management to avoid severe complications. These fractures, while common among young people due to high-energy trauma, also significantly affect the elderly through low-energy falls, necessitating targeted intervention and healthcare resources [1]. Effective treatment includes resuscitation before stabilization, and various surgical techniques such as the use of K-wires and Steinman pins for anatomical reduction and stable fixation [2,3]. A study of 26,357 cases of femoral shaft fractures in adults found that mortality risk was elevated by factors such as advancing age, the presence of other medical conditions, and other injuries, particularly those affecting the thoracic organs, head, and abdomen [4]. Obese patients exhibited higher rates of post-trauma complications compared to those with a normal body mass index [5]. Elderly patients with fall-induced femoral fractures had mortality associated with age, end-stage renal disease (ESRD), and respiratory complications [6]. Although many risk factors have been identified, the overall 30-day mortality rate for patients with proximal femoral fractures ranges from approximately 7.7% to 10.8% [7,8], and delays in surgery beyond 12 to 48 h significantly increase the risk of 30-day mortality [8]. Hence, the prompt identification of patients with a high risk of mortality appears to be a crucial clinical concern in the emergency department.

Hyperglycemia is a frequent physiological response to trauma and stress, and it has been related to increased mortality and morbidity in many medical circumstances [9,10]. After injury, a considerable proportion of hospitalized patients, including 20–30% of trauma patients, have blood glucose levels exceeding 200 mg/dL, with the majority having glucose levels surpassing 150 mg/dL [11]. Richards et al. [12] found that hyperglycemia was an additional contributory factor for surgical site infections in orthopedic trauma patients who did not have a history of diabetes. They discovered that patients with high blood sugar levels around the time of surgery had a significantly greater rate of surgical-site infections. Furthermore, Ay et al. [13] studied the effects of hyperglycemia on rat cortical bone and discovered that it reduced bone quality by increasing osteocyte lacunar density and lowering vascular canal volume. These findings suggest that hyperglycemia can adversely affect bone healing and increase susceptibility to fractures.

The Stress Index (SI), also called the glucose-to-potassium ratio, serves as a metric for metabolic stress and has been examined as a prognostic indicator for death and adverse outcomes in different trauma scenarios [14,15,16,17,18,19,20]. The clinical relevance of the SI is derived from its ability to capture the intricate relationship between hyperglycemia, a marker of the body’s stress response, and variations in potassium levels, which are indicative of cellular injury or systemic metabolic abnormalities [21,22,23,24]. The SI is positioned as a potentially beneficial tool in critical, time-sensitive situations due to its ability to detect physiological changes that are crucial indicators of illness severity and can help guide prompt medical interventions. For instance, in severe traumatic brain injury, a high SI was significantly associated with mortality, underscoring its potential as a predictive marker for trauma severity [17,25]. Additionally, studies have demonstrated the significance of the SI in forecasting death in patients suffering from ischemic stroke and subarachnoid hemorrhage, where changes in potassium-to-glucose levels are correlated with patient outcomes [15,16,18]. Similarly, the SI has been helpful in emergency situations for the early detection of severe trauma and the requirement for damage-control operations [20]. Providing a rapid, objective assessment of the body’s physiological stress response, this simple yet powerful indicator highlights the acute metabolic alterations that arise in extreme medical situations [26]. Hence, by using a retrospective examination of the registered trauma database, this study sought to investigate the correlation and predictive significance between initial SI levels upon presentation at the emergency room and the outcomes of patients with traumatic femoral fractures.

## 2. Materials and Methods

### 2.1. Enrollment of Patients and Research Design

The Chang Gung Memorial Hospital’s Institutional Review Board (IRB) gave approval before the research could begin (approval number 202400311B0). In compliance with IRB regulations, patient consent was waived due to the retrospective nature of the investigation. The study used a retrospective cohort design, analyzing data from adult trauma patients with traumatic femur fractures who were 20 years of age or older and whose codes were S72, S79.0, or S79.1, based on the 10th revision of the International Statistical Classification of Diseases and Related Health Problems (ICD-10). The hospital’s Trauma Registry System provided the data [27], covering the period from 1 January 2009 to 31 December 2022. The study excluded individuals with burns, hanging injuries, drowning incidents, and those with incomplete data. Serum electrolyte levels were determined using baseline laboratory testing performed upon arrival to the emergency room. The SI was derived by dividing the patient’s blood glucose (mg/dL) by their potassium level (mEq/L). The research methodology involved recording all retrieved cases’ basic data, including age, sex, medical history, location and mechanism of injury, laboratory data from blood draws, Glasgow Coma Scale (GCS), Injury Severity Score (ISS), and in-hospital mortality.

### 2.2. Statistical Analysis

This study employed Chi-square testing to assess categorical variables, reporting outcomes as odds ratios (ORs) accompanied by 95% confidence intervals (CIs). Continuous variables were reported as either the mean plus or minus the standard deviation, or as the median with the interquartile range (IQR), depending on whether their distribution followed a normal or non-normal pattern, respectively. The most optimal cut-off value of the SI for forecasting death was discovered by calculating the Area Under the Curve (AUC) of the Receiver Operating Characteristic (ROC) curve by the Youden index [28], which maximizes both sensitivity and specificity in the prediction of outcome. Subsequently, patients were classified according to the SI cut-off in order to determine their risk of death. Adjusted odds ratios (AORs) were then computed for variables such as comorbidities, GCS, and ISS. The selection of these variables was based on their established influence on mortality in patients with severe femoral fractures [4,29,30]. SPSS Windows software, produced by International Business Machines Corporation (IBM), version 23, was utilized for the statistical analysis. A *p*-value of less than 0.05 indicates statistical significance.

## 3. Results

### 3.1. The Study Cohort and Patient Enrollment

**Figure 1:**
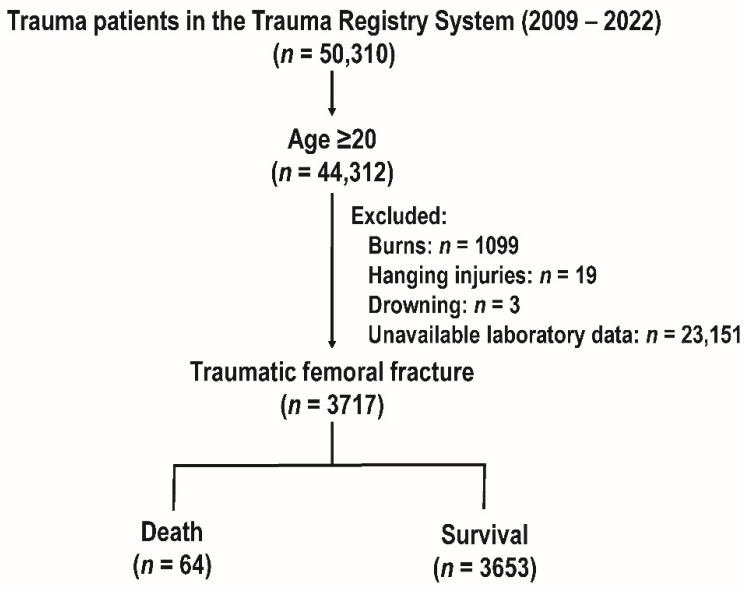
Enrollment process of adult patients with traumatic femoral fractures.

The study focused on individuals with traumatic femur fractures who were 20 years of age or older and examined trauma patients from the Trauma Registry System between 2009 and 2022. At the beginning, there were 50,310 patients in the registry. Upon implementing the age ≥20 inclusion criterion, 44,312 patients remained in the dataset. The focus was further reduced to 3717 patients with traumatic femoral fractures by excluding patients with burns (*n* = 1099), hanging injuries (*n* = 19), drowning (*n* = 3), and patients with inadequate laboratory data (*n* = 23,151). A total of 3717 patients made up the final group, of which 3653 survived and 64 died (Figure 1).

### 3.2. Patient Characteristics

Table 1 reveals that deceased patients were relatively balanced between males (48.4%) and females (51.6%), compared to survivors who had a higher proportion of females (58.4%) than males (41.6%). The average age of deceased patients was slightly younger (66.6 vs. 68.3 years), though this difference was not statistically significant (*p* = 0.491). Both the SI (53.1 vs. 41.6, *p* < 0.001) and blood glucose levels (199.3 vs. 159.0 mg/dL, *p* < 0.001) were considerably higher in the deceased patients. Regarding comorbidities, there were no significant differences in the rates of cerebrovascular accident (CVA), hypertension (HTN), coronary artery disease (CAD), congestive heart failure (CHF), diabetes mellitus (DM), or ESRD between deceased and surviving patients. Deceased patients had a significantly lower GCS score (median 11 vs. 15, *p* < 0.001) and a higher ISS (median 21 vs. 10, *p* < 0.001). Specifically, deceased patients were much more likely to have severe injuries (ISS ≥ 25, 39.1% vs. 4.4%, *p* < 0.001). Hospital stays were longer for deceased patients compared to survivors (16.2 vs. 10.1 days, *p* < 0.001).

### 3.3. The SI’s Mortality Prediction Accuracy

**Figure 2:**
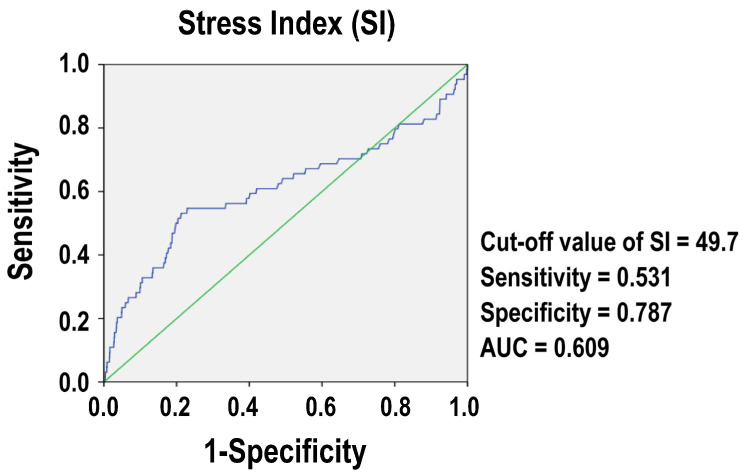
Performance characteristics of the Stress Index (SI) for predicting mortality. The blue line represents the plotting of a Receiver Operating Characteristic (ROC) curve of stress index (SI), while the green line represents a typical diagnostic line with Area Under the Curve (AUC) equal to 0.5.

The SI’s performance features as a prediction tool for evaluating mortality outcomes are shown in Table 2. The ideal cut-off value for the SI to ascertain the mortality result was found to be 49.7 (Figure 2). At this point, the SI’s specificity is 78.7% and its sensitivity is 53.1% for identifying patients who are at danger of passing away. With an AUC of 0.609, the SI is not very good at differentiating between patients with traumatic femur fractures and those without them.

### 3.4. Comparative Evaluation of the Group of Patients Split by the Optimal Cut-Off SI Value

The study contrasts trauma patients with an SI of at least 49.7 with those with a lower index, based on the ideal cut-off value of 49.7. Males had a lower SI and females a greater SI (male OR, 0.82; female OR, 1.22; *p* = 0.014), indicating significant sex differences. The two groups’ ages did not differ much from one another. In comparison to a lower SI, a higher SI was linked to increased incidences of DM (OR 5.96, *p* < 0.001) and HTN (OR 1.38, *p* < 0.001). Significantly longer hospital admissions (12.8 vs. 9.5 days, *p* < 0.001) and greater mortality rates (4.2 vs. 1.0%, *p* <0.001) were seen in patients with higher SIs. When comorbidities, GCS, and ISS were taken into account, the AOR for mortality among individuals with a high SI was substantially higher than that of individuals with a low SI (AOR 2.05, 95%CI 1.14–3.67, *p* = 0.016).

## 4. Discussion

In the present study, although potassium levels did not vary between the deceased and surviving patients, the fatal patients demonstrated significantly elevated blood glucose levels and SIs compared to the survivors. Upon dividing the patients based on the optimal SI cut-off value of 49.7, it was seen that a higher SI was associated with longer hospital admissions and approximately double the adjusted mortality rate compared to those with a lower SI.

The linkage between hyperglycemia and systemic complications has been well recognized in trauma patients. Previous research has demonstrated that admission hyperglycemia is related to mortality in trauma patients with femoral fractures [31]. For example, Rau et al. [31] discovered that patients with stress-induced hyperglycemia or diabetic hyperglycemia had 9.8 times and 5.8 times, respectively, higher death rates compared to patients who did not have hyperglycemia. Comparatively, this study found that deceased patients had higher blood glucose levels (199.3 vs. 159.0 mg/dL, *p* < 0.001) compared to survivors, aligning with previous studies that emphasize the critical role of hyperglycemia in determining the outcomes of traumatic patients.

Potassium levels and their disturbances are critical factors that impact the clinical outcomes of trauma patients [32,33,34]. In 50–68% of trauma patients, hypokalemia is a prevalent occurrence that is frequently associated with the body’s acute stress response [32]. Shi et al. [35] investigated the frequency and therapeutic relevance of potassium, sodium, and calcium electrolyte imbalances in elderly hip fracture patients during surgery and discovered hypokalemia in 32.9% of femoral neck fracture patients, indicating a significant prevalence of potassium abnormalities. Interestingly, the patients who sustained a femoral fracture may have been suffering not only from hypokalemia, but also hyperkalemia, both of which are deleterious to a patient’s health. A high plasma potassium level was associated with 3-month mortality from the blood tests taken on admission in 792 hip fracture patients [36]. Multivariate logistic regression analysis revealed high plasma potassium levels present as an independent risk factor for preoperative asymptomatic pulmonary embolism (OR = 12.9; 95% CI, 1.06–157.30) in elderly patients with hip fractures [37]. Maeda et al. [38] described a case in which an obese patient with insulin-resistant diabetes had deadly ventricular tachycardia as a result of hyperkalemia after femoral fracture surgery. This emphasizes the crucial role of potassium leaking from injured muscles, which causes hyperkalemia and serious cardiac consequences. In this study, the predictive accuracy for mortality is relatively low (AUC = 0.609) in patients with traumatic femoral fracture compared to those with aneurysmal subarachnoid hemorrhage (AUC = 0.747), as reported by Jung et al. [15], severe traumatic brain injury (AUC = 0.777), as reported by Zhou et al. [25], and thoracoabdominal blunt trauma (AUC = 0.854), as reported by Turan et al. [39]. Additionally, the SI has shown diagnostic significance (AUC = 0.733) in separating large from non-massive pulmonary embolism [14]. We speculate that the heterogenous existence of hypokalemia and hyperkalemia, as well as their different effects on increased mortality, may be one of the reasons for the relative lower predictive performance of the SI in traumatic femoral fracture than in other disorders.

Therefore, the changes in serum glucose-to-potassium ratio in patients with traumatic femoral fractures are influenced by several molecular mechanisms. Trauma induces a stress response characterized by the release of catecholamines and glucocorticoids, which increase hepatic glucose production and reduce insulin sensitivity, leading to hyperglycemia [40]. Trauma and the associated inflammatory response can cause potassium to shift from the intracellular to the extracellular space, resulting in hyperkalemia. The release of cytokines such as IL-6 promotes gluconeogenesis and insulin resistance, while elevated adrenocorticotropic hormone (ACTH) levels following trauma further exacerbate hyperglycemia by stimulating cortisol production [41]. Trauma can also lead to ischemic events, affecting cellular metabolism and contributing to alterations in serum glucose and potassium levels. Additionally, hypokalemia, often observed in trauma patients, may result from a catecholamine surge that causes intracellular potassium shifts via the beta-2 adrenergic stimulation of the Na+-K+ pump [42,43]. This phenomenon is especially pronounced in younger patients and those with severe injuries, leading to significant morbidity and requiring careful management to avoid complications [44].

Some other factors may have contributed to the SI’s relatively low predictive value in this investigation. First, the variability of the patient group, which encompassed both traumatic injuries in younger individuals with high-energy impact and falls in the elderly with low-energy impact, may have resulted in variable physiological reactions and metabolic stressors, reducing SI accuracy. Second, the SI is impacted by glucose and potassium levels, which can fluctuate quickly after injury and during first resuscitation, resulting in variability depending on when blood samples are collected. This variability may be less prominent in circumstances such as subarachnoid hemorrhage or traumatic brain injury, where the metabolic response is more uniform [19,25,45]. These factors, taken together, may explain the observed disparities in the SI’s predictive value when compared to other research with more homogeneous populations. Despite the constraints, this study discovered that adjusted mortality is considerably greater for patients with elevated SIs, with an AOR of 2.05. This shows that the association between heightened SI and increased mortality highlights its potential use as an early risk classification tool in clinical settings, allowing for more prompt and targeted interventions.

There were several limitations in this study. First, it is a retrospective, single-center study, which may create selection biases and limit the findings’ applicability to other contexts or groups. Second, the accuracy of the SI measurements may be impacted by the study’s dependence on a single time point for the collection of blood samples following injury, which may not adequately capture the dynamic changes in glucose and potassium levels. Additionally, a biased assessment of mortality outcomes may have resulted from the study’s failure to include patients who were declared fatal at the scene of the accident or upon arrival at the emergency department. Furthermore, the condition of resuscitation and whether fluid was challenged before arrival to the emergency room were unknown. Finally, the study assumes that a variety of physicians’ interventions and surgical management methods had a uniform effect on these patients. The combination of these characteristics emphasizes the necessity for prospective, multi-center research to define standardized cut-off values for wider clinical applications and evaluate the predictive ability of the SI.

## Figures and Tables

**Table 1:** Patient and injury characteristics of the deceased and surviving patients.

Variables	Death *n* = 64	Survival *n* = 3653	OR (95%CI)	*p*
Sex				0.272
Male, *n* (%)	31 (48.4)	1520 (41.6)	1.32 (0.80–2.16)	
Female, *n* (%)	33 (51.6)	2133 (58.4)	0.76 (0.46–1.24)	
Age, years (mean ± SD)	66.6 ± 18.7	68.3 ± 18.7	-	0.491
Stress Index	53.1 ± 28.0	41.6 ± 17.6	-	<0.001
Sugar (mg/dL)	199.3 ± 102.8	159.0 ± 67.3	-	<0.001
Potassium (mEq/L)	3.9 ± 1.0	3.9 ± 0.6	-	0.818
Comorbidities				
CVA, *n* (%)	2 (3.1)	371 (10.2)	0.29 (0.07–1.17)	0.063
HTN, *n* (%)	34 (53.1)	1843 (50.5)	1.11 (0.68–1.83)	0.672
CAD, *n* (%)	7 (10.9)	271 (7.4)	1.53 (0.69–3.39)	0.289
CHF, *n* (%)	1 (1.6)	62 (1.7)	0.92 (0.13–6.74)	0.934
DM, *n* (%)	23 (35.9)	1009 (27.6)	1.47 (0.88–2.46)	0.141
ESRD, *n* (%)	3 (4.7)	131 (3.6)	1.32 (0.41–4.27)	0.639
GCS, median (IQR)	11 (6–15)	15 (15–15)	-	<0.001
3–8, *n* (%)	23 (35.9)	59 (1.6)	34.17 (19.29–60.53)	<0.001
9–12, *n* (%)	4 (6.2)	110 (3.0)	2.15 (0.77–6.01)	0.136
13–15, *n* (%)	37 (57.8)	3484 (95.4)	0.07 (0.04–0.11)	<0.001
ISS, median (IQR)	21 (9–34)	10 (9–9)	-	<0.001
1–15, *n* (%)	33 (51.6)	3355 (91.8)	0.10 (0.06–0.16)	<0.001
16–24, *n* (%)	6 (9.4)	138 (3.8)	2.64 (1.12–6.21)	0.021
≥25, *n* (%)	25 (39.1)	160 (4.4)	13.99 (8.27–23.69)	<0.001
Hospital stay (days)	16.2 ± 20.7	10.1 ± 9.3	-	<0.001

CAD, coronary artery disease; CHF, congestive heart failure; CVA, cerebrovascular accident; CI, confidence interval; DM, diabetes mellitus; ESRD, end-stage renal disease; GCS, Glasgow Coma Scale; HTN, hypertension; IQR, interquartile range; ISS, Injury Severity Score; OR, odds ratio; SD, standard deviation.

**Table 2:** Comparative analysis of patients with high and low Stress Index (SI) based on the optimal cut-off value of 49.7.

SI				
Variables	≥49.7 *n* = 811	<49.7 *n* = 2906	OR (95%CI)	*p*
Sex				0.014
Male, *n* (%)	308 (38.0)	1243 (42.8)	0.82 (0.70–0.96)	
Female, *n* (%)	503 (62.0)	1663 (57.2)	1.22 (1.04–1.43)	
Age, years (mean ± SD)	68.8 ± 16.4	68.1 ± 19.3	-	0.295
Comorbidities				
CVA, *n* (%)	86 (10.6)	287 (9.9)	1.08 (0.84–1.40)	0.542
HTN, *n* (%)	460 (56.7)	1417 (48.8)	1.38 (1.18–1.61)	<0.001
CAD, *n* (%)	63 (7.8)	215 (7.4)	1.05 (0.79–1.41)	0.723
CHF, *n* (%)	13 (1.6)	50 (1.7)	0.93 (0.50–1.72)	0.819
DM, *n* (%)	475 (58.6)	557 (19.2)	5.96 (5.04–7.05)	<0.001
ESRD, *n* (%)	29 (3.6)	105 (3.6)	0.99 (0.65–1.50)	0.960
GCS, median (IQR)	15 (15–15)	15 (15–15)	-	<0.001
3–8, *n* (%)	44 (5.4)	38 (1.3)	4.33 (2.79–6.73)	<0.001
9–12, *n* (%)	34 (4.2)	80 (2.8)	1.55 (1.03–2.33)	0.036
13–15, *n* (%)	733 (90.4)	2788 (95.9)	0.40 (0.30–0.54)	<0.001
ISS, median (IQR)	9 (9–9)	9 (9–9)	-	<0.001
1–15, *n* (%)	679 (83.7)	2709 (93.2)	0.37 (0.30–0.47)	<0.001
16–24, *n* (%)	50 (6.2)	94 (3.2)	1.97 (1.38–2.80)	<0.001
≥25, *n* (%)	82 (10.1)	103 (3.5)	3.06 (2.27–4.14)	<0.001
Hospital stay (days)	12.8 ± 13.6	9.5 ± 8.0	-	<0.001
Mortality, *n* (%)	34 (4.2)	30 (1.0)	4.20 (2.55–6.90)	<0.001
AOR of mortality *	-	-	2.05 (1.14–3.67)	0.016

AOR, adjusted odds ratio; CAD, coronary artery disease; CHF, congestive heart failure; CVA, cerebrovascular accident; CI, confidence interval; DM, diabetes mellitus; ESRD, end-stage renal disease; GCS, Glasgow Coma Scale; HTN, hypertension; IQR, interquartile range; ISS, Injury Severity Score; OR, odds ratio; SD, standard deviation; * mortality adjusted by comorbidities, GCS, and ISS.

## Data Availability

The de-identified data presented in this study are available on request from the corresponding author. The original data are not publicly available due to privacy concern.
